# Silicon Fresnel Zone Plate Metalens with Subwavelength Gratings

**DOI:** 10.3390/s23084137

**Published:** 2023-04-20

**Authors:** William Fraser, Winnie N. Ye

**Affiliations:** Silicon Micro/NanoPhotonics Group, Carleton University, Ottawa, ON K1S 5B6, Canada

**Keywords:** metalens, silicon photonics, subwavelength grating, Fresnel zone plate

## Abstract

Metalenses are planar optical components that have demonstrated immense potential for integrated optics. In particular, they are capable of high-efficiency subwavelength focusing without the bulkiness of traditional lenses. Dielectric metalenses operating in the C-band typically employ relatively tall, amorphous silicon structures arranged in a periodic array. Phase control spanning from 0 to 2π is accessed by varying the geometry of these scattering structures. The full 2π phase range is necessary to impose a hyperbolic focusing phase profile, but this is difficult to achieve without custom fabrication practices. In this work, we propose a binary phase Fresnel zone plate metalens designed for the standard 500 nm silicon-on-insulator platform. Our design uses subwavelength gratings with trapezoidal segmentation to form concentric rings. The effective index of the grating is set with the duty cycle using a single full-etch step to form the binary phase profile of the zone plate. The metalens design can be easily tuned to achieve longer focal lengths at different wavelengths. It offers a simple platform for high-throughput wavelength-scale focusing elements in free-space optics, including for microscopy and medical imaging.

## 1. Introduction

In recent years, the integration of imaging systems on photonic integrated circuits (PICs) has driven the development of wavelength-scale optical components. Metasurfaces [1,2,3,4,5] leverage the wavefront-shaping capabilities of subwavelength structures to achieve a wide range of optical effects. Their small footprint and compatibility with complementary metal–oxide–semiconductor (CMOS) fabrication practices enables simple integration with photonic systems [4]. All-dielectric focusing metalenses have shown immense potential at datacom and telecom wavelengths. This is largely due to their low absorption loss in the visible and near-infrared spectral regions compared to plasmonic antennas [3]. A significant amount of effort has been invested in the development of large-numerical-aperture (NA) and high-efficiency metalenses that produce a sharp focal spot. Amorphous silicon metalenses with a 940 nm thickness are presented in [6] with a numerical aperture of 0.97 operating at 1550 nm. Focusing efficiencies of 42% and 82% and focal spot sizes of 0.57 λ and 2.4 λ were reported at focal lengths of 50 µm and 500 µm, respectively. In [7], diffraction-limited focusing efficiencies of 86%, 73%, and 66% were reported for TiO_2_ metalenses with an NA of 0.8 operating at 405 nm, 532 nm, and 660 nm, respectively. Later, ultrahigh numerical apertures of 0.98 in air and 1.48 in oil were achieved at 532 nm with a focal length of 5.1 µm using crystalline silicon on sapphire [8]. Focusing efficiencies of 67% and 48% with focal spot sizes of 0.5 λ and 0.4 λ were reported in air and oil, respectively, with a bandwidth of 274 nm. 

Achromatic focusing has also been an important theme in metasurface research, aiming to eliminate the intrinsic chromatic aberrations of diffractive optics. Solutions for achromatic focusing of discrete wavelengths spanning a specific spectral band, as well as broadband focusing schemes, have been reported. In [9], a one-dimensional, amorphous silicon grating metalens was shown to be capable of focusing light at three discrete wavelengths in the near-infrared region through phase dispersion compensation. However, the numerical aperture was only 0.05, with focusing efficiencies of 9.8%, 10.3%, and 12.6% at 1300 nm, 1550 nm, and 1800 nm, respectively. The first demonstration of metalens-assisted continuous band imaging in the visible spectrum was presented in [10] with a silicon nitride metalens that produced an extended depth of focus over the 400 nm to 700 nm spectral window with a numerical aperture of 0.45 and focusing efficiency of up to 63%. The lens was paired with a single digital filter to achieve full-colour imaging. An inverse-designed achromatic metalens with a large numerical aperture was demonstrated in [11] using TiO_2_. An NA of 0.99 was reported, with an average focusing efficiency of 27% and focal spot size of 0.85 λ over the bandwidth from 450 nm to 700 nm. The next year, another TiO_2_ metalens was demonstrated in [12] with a focusing efficiency of up to 88.5% and NA between 0.1 and 0.24 for the 650 nm to 1000 nm clinical imaging window. Finally, a near-diffraction-limited silicon nitride metalens with an ultrawide bandwidth of 950 nm spanning the visible and near-infrared spectral bands and focusing efficiency of 70% was achieved in [13]. However, the numerical aperture was limited to 0.107. The exponential growth of interest in metasurface research over recent years has led to significant breakthroughs in the compact integration of imaging and sensing systems. In particular, silicon-based metalenses have been demonstrated in a variety of applications operating in the near-infrared spectral region.

A silicon metalens with a focusing efficiency of 68% was implemented in a novel two-dimensional beam-steering platform for ultra-low-power light detection and ranging (LiDAR) systems [14]. An aberration-corrected silicon metalens was demonstrated in [15] with subwavelength focusing capabilities in the 1250 nm to 1370 nm clinical imaging window for high-resolution optical coherence tomography over an extended imaging depth. Silicon metalenses most commonly employ subwavelength arrays of pillars [6,14,15,16,17] or rectangular fins [9,18,19]. The field is strongly confined in these elements and accumulates a phase shift as it propagates through the metasurface [20]. The amount of phase accumulation can be actively adjusted by varying the size of the scatterers to control their effective index [3]. For a typical focusing metalens, the library of available unit cell geometries must span the full range from 0 to 2π. This is necessary to impose a hyperbolic phase profile, which converts incident plane waves into converging spherical wavefronts [1,2]. This requires metalens thicknesses close to the operating wavelength [21], making it impractical for realization in any standard silicon-on-insulator (SOI) platform. Thinner metasurfaces have been developed by coupling to the Mie resonances of the subwavelength structures to form Huygens surfaces [22,23]. This has been demonstrated on the 220 nm SOI platform [23] but requires very precise nanostructure geometries to precisely overlap the electric and magnetic resonances at the operating wavelength. The design process in [23] is quite complicated, with significant limitations in performance. In fact, most metalenses demonstrated around 1550 nm require custom fabrication runs with a layer of amorphous silicon close to 1 µm in thickness [6,14,15,16]. 

Dielectric focusing metalenses based on a binary phase Fresnel zone plate (BPFZP) are capable of breaking the diffraction limit [24,25]. They have also been demonstrated with impressive achromatic focusing abilities. A TiO_2_ BPFZP metalens described in [26] achieved achromatic focusing at 473 nm, 532 nm, and 633 nm, with focal spot sizes as low as 0.51 λ and a numerical aperture of 0.97. However, the focusing efficiency was only 3%. Early implementations of silicon-based Fresnel zone plates (FZPs) all used a silica substrate and required custom fabrication of the lens layers. One design involved etching a thin layer of 50 nm amorphous silicon on a silica substrate [27]; another relied on Si3N4 ring sets on silica, each with a 1.2 mm aperture [28]; an interesting three-dimensional Fresnel lens using polysilicon on glass attached with micro-hinges was shown to form collimated beams at near-infrared [29]. Modern fabrication techniques have significantly improved the performance of silicon Fresnel zone plates. As a result, FZP lenses continue to be a valuable component for the miniaturization of imaging and free-space communication/sensing systems [30,31,32,33,34]. However, these reported FZP lenses employ unique material platforms and require custom fabrication because the lens thickness imposes significant limitations on achieving the necessary phase profile [29]. This limits the design flexibility of FZPs when only specific material thicknesses and etch depths are available, as is commonly the case with public foundries.

In this work, we present the design of a novel SOI-compatible BPFZP metalens with subwavelength focusing capability operating at 1550 nm. Our proposed design can be fabricated on a standard 500 nm SOI wafer with only one single full-etch step and can be easily adapted for other wavelengths and focal distances. It offers a very simple platform for high-throughput wavelength-scale focusing elements in free-space optics. To the best of our knowledge, this is the first ever demonstration of high-efficiency subwavelength focusing with flat optics on a standard SOI platform.

## 2. Materials and Methods

**Figure 1:**
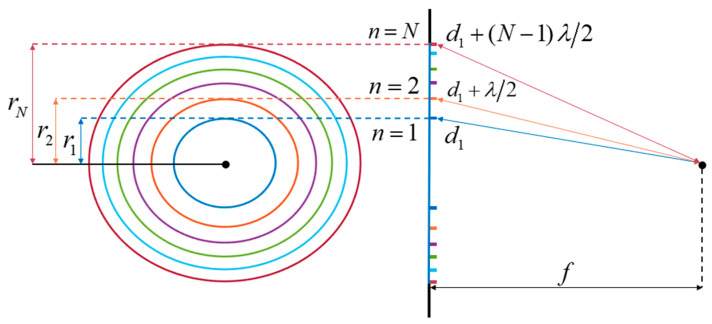
Diagram of the proposed Fresnel zone plate and its associated parameters.

The Fresnel zone plate consists of a series of concentric annular regions, or Fresnel zones, that form a circular aperture. Each zone is identified with a zone number (*n* = 1, 2, …, N) counting out from the center of the aperture and a radius (r_n_). A diagram of the FZP is shown in Figure 1.

Fresnel postulated that the radii of these zones could be set such that the path length difference to a chosen focal point increases by a half-wavelength from one zone to the next [35,36]. The radii that satisfy this condition are simply derived from the Pythagorean theorem, leading to Equation (1) [36]:

(1) $$r_{n}=f\sqrt{\frac{n\lambda }{f}+{\left(\frac{n\lambda }{2f}\right)}^{2}}$$

where λ is the wavelength, *f* is the focal length, and *n* is the Fresnel zone number, as labelled in Figure 1. Since the difference in the optical path length between adjacent zones to the focal point is a half-wavelength, the odd-numbered zones (*n* = 1, 3, …) interfere destructively with the even-numbered zones (*n* = 2, 4, …). Introducing an additional phase shift of π in the path of one set of Fresnel zones compensates for the phase mismatch at the focal point. As a result, the full aperture contributes constructively at the focal point. This concept guides the design of binary phase Fresnel zone plates, which imposes the phase profile of alternating zones with 0 and π relative phase difference to perform high-efficiency focusing [24,26,37].

Conventional metalenses focus light by converting incoming planar wavefronts into spherical waves that converge at a point. Without access to the full phase range from 0 to 2π, the transmitted wavefronts would not be perfectly spherical, and the lens would suffer from spherical aberrations, which diminishes the quality of the focus [2]. A possible solution to this is to limit the size of the lens according to the available phase coverage [24]. However, this places a constraint on the numerical aperture that can be achieved. In contrast, a BPFZP is analogous to a signal rectifier, flipping the sign of contributions from regions in the aperture that would otherwise interfere destructively at the focus. Since the phase profile only takes on values of 0 and π, it is much simpler to implement in silicon with standard SOI platforms.

Removing the dependence on material thickness when imposing the BPFZP phase profile is necessary for flexible integration on the SOI platform. Filling the grooves of the FZP with another material can be undertaken to create a refractive index contrast between adjacent zones. With the correct index contrast, the difference in phase accumulation as the light passes through the materials will be π [24]. This can be undertaken directly in silicon by forming a subwavelength metamaterial in the even-numbered Fresnel zones.

**Figure 2:**
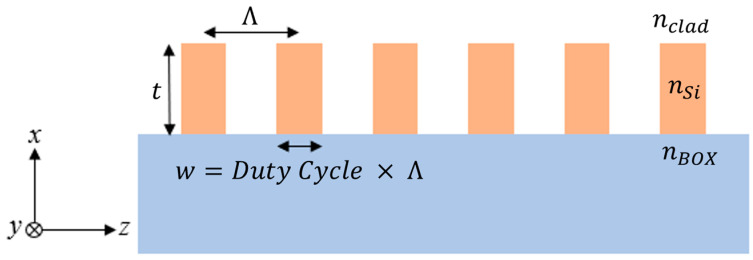
Sideview diagram of *z*-periodic subwavelength grating on SOI.

Subwavelength gratings (SWGs), as depicted in Figure 2, are a periodic arrangement of segments with a pitch below the Bragg threshold [38,39]. SWGs are known for their design flexibility in effective index engineering thanks to their ability to vary the period, Λ, and width, *w*, of the grating segments [38]. This enables effective tuning of the phase accumulation without having to change the thickness, *t*, of the silicon layer. In Figure 2, the individual segments of the SWG are repeated along the z-axis, which forms a one-dimensional SWG that is *z*-periodic [39]. 

When the period of the grating is very small compared to the operating wavelength, light propagates through the structure beyond the diffraction limit. Consequently, the light interacts with the grating as a homogenous medium. The equivalent refractive index is proportional to the indices of the constituent materials, as well as the ratio of *w* to Λ, otherwise known as the duty cycle [38]. When an electromagnetic wave propagates through the structure in the direction perpendicular to the periodicity of the grating (i.e., the *x*-direction in Figure 2), the equivalent refractive index can be approximated with Rytov’s formulas, given as Equations (2) and (3) [40]:

(2) $$n_{\left|\right|}^{2}\approx \frac{w}{\Lambda }n_{Si}^{2}+\left(1-\frac{w}{\Lambda }\right)n_{clad}^{2}$$

(3) $$\frac{1}{n_{⊥}^{2}}\approx \frac{w}{\Lambda }n_{Si}^{-2}+\left(1-\frac{w}{\Lambda }\right)\frac{1}{n_{clad}^{2}}\;$$

where *n_||_* refers to the index experienced by waves polarized in the direction parallel to the grating segments and *n_⊥_* applies to the polarization perpendicular to the grating segments. The parallel and perpendicular polarizations correspond to the *x*- and *z*-directions in Figure 2, respectively, for a wave propagating along the *y*-direction. These equations, which are commonly referred to as the zeroth-order Rytov equations, are only valid when the period of the SWG is significantly smaller than the wavelength in the material. However, this would require a pitch of 100 nm or smaller due to the high refractive index of silicon at 1550 nm. Such a small period is not feasible in modern fabrication practices. For this reason, SWG devices designed to operate at 1550 nm will often employ a pitch in the 300 nm to 400 nm range, which is still small enough to leverage the index engineering capabilities [38]. With these larger pitches, however, the second-order Rytov equations are required to represent a more accurate approximation of the equivalent refractive index [41]:

(4) $$n_{\left|\right|}^{\left(2\right)}=n_{\left|\right|}\sqrt{1+\frac{\pi^{2}}{3}{\left(\frac{n_{eff}\Lambda }{\lambda }\right)}^{2}{\left(1-\frac{w}{\Lambda }\right)}^{2}\frac{w^{2}}{\Lambda^{2}}{\left(\frac{n_{clad}^{2}-n_{Si}^{2}}{n_{eff_{TM}}n_{\left|\right|}}\right)}^{2}}$$

(5) $$n_{⊥}^{\left(2\right)}=n_{⊥}\sqrt{1+\frac{\pi^{2}}{3}{\left(\frac{n_{eff}\Lambda }{\lambda }\right)}^{2}\frac{w^{2}}{\Lambda^{2}}{\left(1-\frac{w}{\Lambda }\right)}^{2}{\left(n_{clad}^{2}-n_{Si}^{2}\right)}^{2}{\left(\frac{n_{\left|\right|}}{n_{eff_{TE}}}\right)}^{2}{\left(\frac{n_{⊥}}{n_{clad}n_{Si}}\right)}^{4}}$$

where *n_eff_^TM^* and *n_eff_^TE^* are the effective indexes of the transverse magnetic (TM) and transverse electric (TE) modes in a slab waveguide with the same thickness as the SWG, respectively.

Subwavelength gratings offer a very simple platform for implementing BPFZPs. The required phase profile can be realized by alternating between non-etched silicon regions for odd-numbered Fresnel zones and SWG rings for even zones. The duty cycle of the SWGs can be chosen such that the difference in refractive index between the etched and non-etched regions creates a relative phase difference of π in the near field. According to Rytov’s formulas, this equivalent index is directly related to the angle between the SWG segments and the direction of the light’s polarization. Since the SWGs in our design have a circular formation, the orientation of the individual segments is not uniform across the surface of the metalens. Therefore, the equivalent refractive index will vary greatly for a given polarization if the duty cycle is held constant. This leads to two possible designs: (i) a polarization-independent configuration with a constant SWG duty cycle around the rings and (ii) a polarization-specific design with a varying duty cycle to accommodate for the orientation of the SWG segments.

### 2.1. Polarization-Independent Binary Phase Fresnel Zone Plate

**Figure 3:**
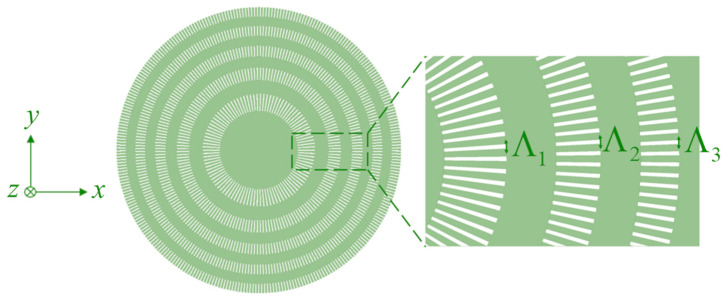
Top-view diagram of our proposed polarization-independent binary phase Fresnel zone plate using subwavelength gratings (SWGs). The duty cycles of the SWGs are constant at a given zone.

A top-view diagram of the polarization-independent SWG-based BPFZP proposed in this work is shown in Figure 3. For the polarization-independent design, the duty cycle can be set based on either of the Rytov equations. If Equation (5) is used, a beam polarized along the *x*-axis would sense an equivalent index near the desired refractive index in the top and bottom quadrants of the lens where the polarization is perpendicular or near-perpendicular to the SWG segments. The opposite would occur for a beam polarized along the *y*-axis. As a result, both polarizations will acquire the same phase profile, rotated by π/2 relative to each other, when transmitted through the lens.

The lens was designed for a focal length of 10 µm at an operating wavelength of 1.55 µm and contains ten Fresnel zones. The number of zones and 10 µm focal length were chosen to reduce the size of the lens for ease of simulation while maintaining a large numerical aperture to produce a small focal spot. With this short focal length, it is possible to directly capture the full propagation of the transmitted beam from the lens to the focal spot with a relatively high resolution rather than depending on the coarse far-field projections that would be required for a lens with a focal length in the order of millimeters. A nominal grating period of 300 nm was chosen to ensure a reasonable minimum feature size while still operating in the subwavelength regime [38]. The actual period differs slightly for each SWG and is set according to the circumference at the center of the ring such that there is an integer number of equal periods in each zone. The individual segments are trapezoidal, with the inner edge being shorter than the outer edge to maintain an equal spacing from the inner radius to the outer radius, as shown in the inset of Figure 3. An approximate duty cycle was selected using Equation (5) and optimized to 66.5%. This translates to a minimum feature size of 100.5 nm, which is feasible with standard lithography techniques. The optimization was performed using rigorous 3D finite-difference time-domain (FDTD) simulations in the Ansys Lumerical FDTD simulation suite. The FDTD algorithm was selected over the often faster rigorous coupled-wave analysis (RCWA) method due to the lack of periodicity in the plane transverse to the direction of propagation. FDTD simulations are also better suited for spectral analysis. The lens has a diameter of 29.33 µm and a numerical aperture of 0.83. The short focal length makes this metalens particularly useful for microscopy [31] but the concept can easily be scaled up to larger geometries for applications in medical imaging [14] and spatial multiplexing [42]. 

### 2.2. Polarization-Specific Binary Phase Fresnel Zone Plate

**Figure 4:**
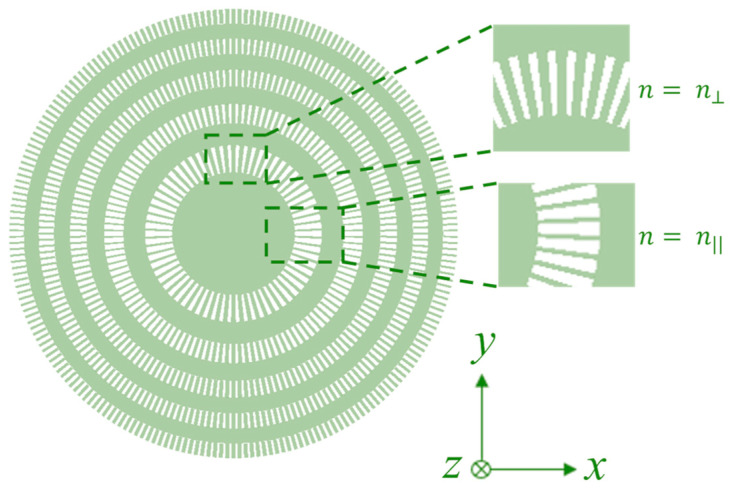
Top-view diagram of our proposed polarization-specific binary phase Fresnel zone plate using subwavelength gratings (SWGs). Note that the duty cycles of the SWGs in each ring depend on the orientation of the segments.

A schematic of the polarization-specific SWG-based BPFZP is shown in Figure 4. This design is optimized for light that is linearly polarized along the *x*-axis. The duty cycle of the SWG rings is varied as a function of the SWG segment orientation. This ensures a more uniform equivalent refractive index around the segmented ring. Rotating the lens by π/2 would give the same result for light polarized along the vertical axis. 

At the top and bottom of the rings, the equivalent index follows Equation (5), whereas on the sides of the rings, the equivalent index follows Equation (4). Therefore, the duty cycle of the SWGs is higher at the top and bottom compared to the sides, as shown in the insets of Figure 4. The duty cycle is varied linearly from the minimum to the maximum value in angular intervals of π/2 around the rings. The duty cycles were optimized using 3D FDTD simulations for each ring individually and vary between 31.5% and 66.5%. In this design, a nominal SWG period of 400 nm is used to ensure a minimum feature size above 100 nm with the lower duty cycle.

## 3. Results

In the simulations, the lens was illuminated with a linearly polarized Gaussian beam propagating through the lens in the *z*-direction to mimic the conditions for free-space focusing. For both designs, the focusing properties of the lens under the influence of light polarized along both the *x*- and *y*-axis were investigated.

### 3.1. Polarization-Independent BPFZP Design

**Figure 5:**
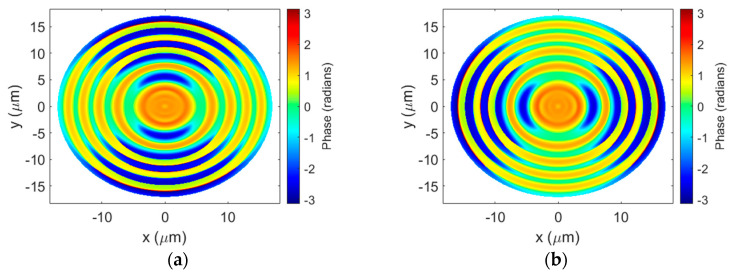
Near-field phase distributions of the (**a**) *x*-polarized and (**b**) *y*-polarized transmitted beams. The BPFZP is illuminated with a linearly polarized Gaussian beam propagating through the lens in the *z*-direction.

The near-field phase distributions of the transmitted *x*- and *y*-polarized beams are shown in Figure 5a,b, respectively. The difference in phase accumulation between the solid rings and the SWG rings is clear. As expected, the phase is constant in the unetched regions but varies in the SWGs with the angle of the grating segments. The relative near-field phase distribution of the *y*-polarized light is the same as that of the *x*-polarized light rotated by π/2 due to the rotational symmetry of the structure. This effect is what gives rise to the polarization-insensitive response of the lens.

**Figure 6:**
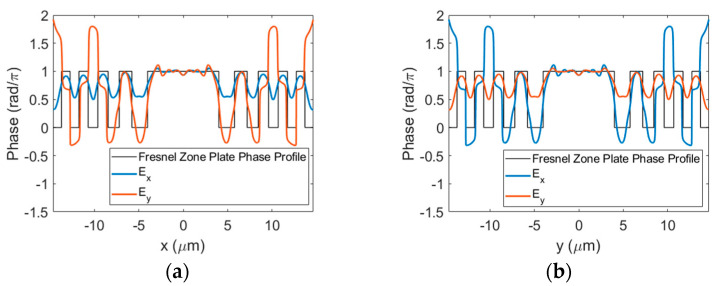
Cross-sections of the near-field phase along the (**a**) horizontal and (**b**) vertical axes through the center of the BPFZP zone plate.

The cross-sections of the near-field phase taken horizontally and vertically through the center of the lens for both polarizations are plotted in Figure 6a,b, respectively. Since the duty cycle was optimized based on Equation (5), we see better agreement between an ideal BPFZP phase profile and the phase of the *y*-polarized light along the horizontal axis where the SWG segments are perpendicular to the *y*-direction. In contrast, the phase difference between the SWG rings and the unetched regions is much smaller for the *x*-polarized light since the polarization is parallel to the segments. Similarly, the phase of the *x*-polarized light follows the BPFZP profile more closely where the SWG segments are vertical and the *y*-polarized light exhibits a smaller relative phase difference. 

From Figure 6a,b, we can note that the relative phase profile does not completely match that of an ideal BPFZP on either axis for both linear polarizations. Since the duty cycle of the SWG rings is constant, there is a trade-off between the accuracy of the phase profile along one axis compared to the other for a given polarization. For example, if the duty cycle were selected such that the near-field phase of the *x*-polarized light perfectly matched the ideal BPFZP profile along the vertical axis of the lens, the overlap would be reduced on the horizontal axis. This can degrade the performance of the lens because less of the surface contributes constructively at the focal point. Balancing this trade-off was the primary goal during the optimization of the design.

**Figure 7:**
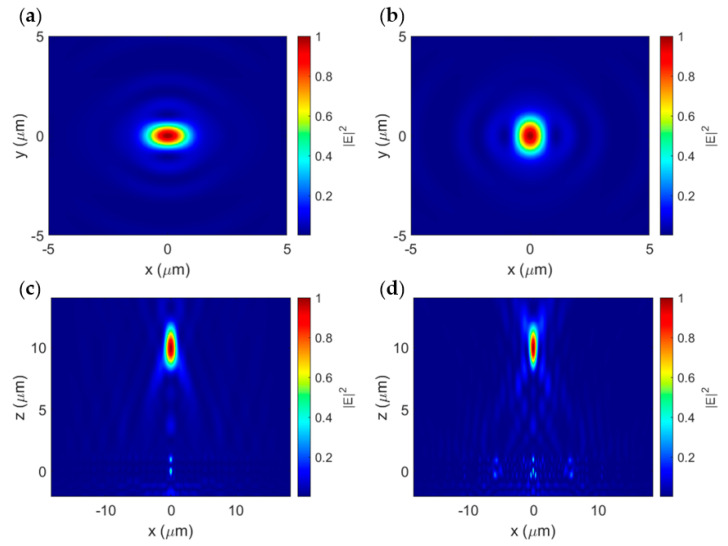
Normalized electric field intensity distribution of (**a**) focal plane for *x*-polarized light, (**b**) focal plane for *y*-polarized light, (**c**) propagation cross-section along focal spot major axis, and (**d**) propagation cross-section along focal spot minor axis.

The normalized electric field intensity distributions at the focal plane for the *x*-polarization and *y*-polarization are shown in Figure 7a,b, respectively. The focal spot forms an ellipse with its major axis parallel to the direction of the linear polarization [43]. Once again, the responses to both linear polarizations are identical other than the π/2 rotation, with full widths at half-maximum (FWHMs) of 0.84 λ and 0.53 λ along the major and minor axes of the focal spot, respectively. Cross-sections of the normalized electric field intensity distribution of the transmitted beam taken along the major and minor axes of the focal spot are presented in Figure 7c,d, respectively, showing the simulated focal length of 9.86 µm, which is only 1.4% off from the nominal 10 µm focal length.

**Figure 8:**
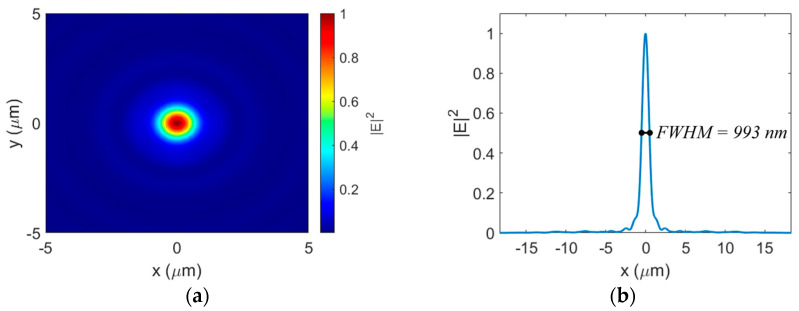
(**a**) Normalized electric field intensity distribution at the focal plane with circularly polarized light. (**b**) Focal spot cross-section for circular polarization.

A circular focal spot is produced when the incident light has components polarized in both directions. Figure 8a,b show the normalized electric field intensity distribution at the focal plane and a slice taken across the focal spot, respectively, for a circularly polarized incident beam. In this case, the transmitted beam forms a circular focal spot with an FWHM of 0.64 λ. This is near the diffraction limit of 0.5 λ/NA = 0.6 λ [26]. This subwavelength focusing ability is a major advantage compared to the only other reported metalens fabricated on a 500 nm SOI platform [17], which offered a focusing beam spot of 11 µm.

Here, the focusing efficiency is defined as the ratio between the power focused into a circular region with a radius three times the FWHM and the total power transmitted by the lens [6]. For the circular polarization, the metalens achieved a focusing efficiency of 39.2%, while for the linear polarizations, the focusing efficiency was 35.9% based on the minor axis FWHM of the elliptical focal spot. Although this focusing efficiency is not as high as that in traditional meta atom-based hyperbolic metalenses [6,8,10,12,13,14], it is comparable, if not better, than other monochromatic FZP-based metalenses [26,44]. Our proposed metalens design is the first of its kind fabricated on a 500 nm SOI platform with a focal spot size in the subwavelength range.

**Figure 9:**
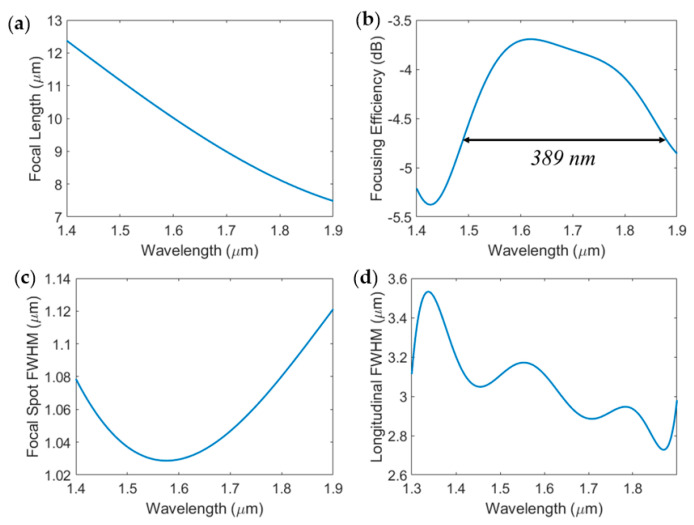
Wavelength dependence of (**a**) focal length, (**b**) focal spot transversal FWHM, (**c**) focal spot longitudinal FWHM, and (**d**) the focusing efficiency of our proposed BPFZP with uniform SWG duty cycle.

The focal length of the lens can be shifted by wavelength tuning, as plotted in Figure 9a. The wavelength dependency of the focusing efficiency is plotted in Figure 9b. The 1 dB bandwidth was calculated to be 389 nm centered at 1.68 µm. The lens maintains a subwavelength focal point transverse FWHM and relatively stable (<1 µm variation) longitudinal FWHM over the entire bandwidth, as shown in Figure 9c,d, respectively. These properties can be of particular interest for three-dimensional imaging where the imaging plane is wavelength-tunable.

The 1 dB bandwidth of our proposed design is slightly better, or comparable to, previously demonstrated achromatic hyperbolic and FZP-based focusing metalenses [9,12,15,26,45]. This is because introducing any amount of relative phase shift between adjacent Fresnel zones will give rise to the focusing effect. Although the structure was optimized to overlap the ideal BPFZP profile at a specific wavelength, the equivalent refractive index of the SWGs is still lower than that of the solid rings at all wavelengths. As a result, the contributions from the even-numbered zones are not completely out of phase at the focal point, even for wavelengths other than 1.55 µm. Therefore, the destructive interference is reduced and the positive contributions from the unetched Fresnel zones dominate over the 389 nm 1 dB bandwidth. A comparison of our results to similar metalenses reported in the literature is presented in Table 1. Our design provides a reasonable focusing efficiency and large bandwidth while maintaining a large numerical aperture. 

### 3.2. Polarization-Specific BPFZP Design

**Figure 10:**
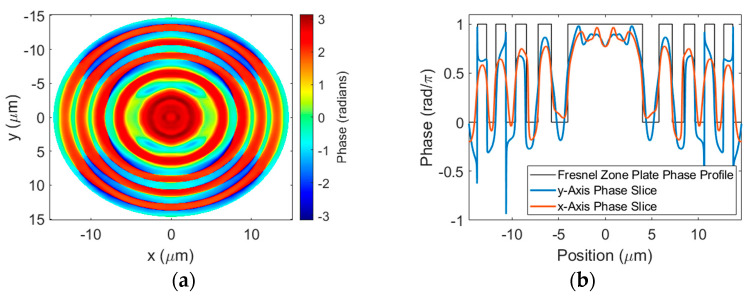
(**a**) Near-field phase distribution of transmitted beam with *x*-polarization. (**b**) Cross-sections of near-field phase taken along the vertical and horizontal axes of the lens with overlay of ideal BPFZP profile.

The near-field phase distribution of the transmitted beam with *x*-polarization is shown in Figure 10a. Although there is still some non-uniformity of the phase in the SWG regions, comparing to Figure 5a, we see that the variation in the phase is less pronounced. Cross-sections along the vertical and horizontal axes of the lens are plotted in Figure 10b and show improved overlap with the ideal BPFZP phase profile for both slices compared to Figure 6a.

**Figure 11:**
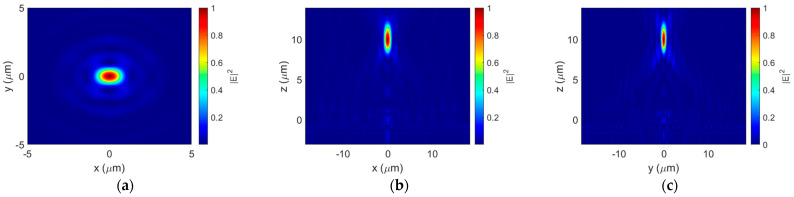
Normalized electric field intensity distributions: (**a**) at the focal plane, (**b**) of the propagation cross-section taken along the major axis of the focal spot, and (**c**) of the propagation cross-section taken along the minor axis of the focal spot.

The normalized electric field distribution at the focal plane is shown in Figure 11a. Cross-sections of the transmitted beam’s normalized electric field distribution across the major and minor axes of the focal spot are shown in Figure 11b,c, respectively. The elliptical focal point has an FWHM of 0.75 λ along the major axis and 0.53 λ along the minor axis of the ellipse, with a focusing efficiency of 45.2% at the 10 µm focal length. Therefore, varying the duty cycle to compensate for the change in equivalent refractive index improved the focusing efficiency while maintaining the subwavelength focal point dimensions. It also aligned the focal spot with the desired focal length. On top of that, although this performance was demonstrated for *x*-polarized light, simply rotating the lens by π/2 would result in the same focusing ability for the *y*-polarization. 

**Figure 12:**
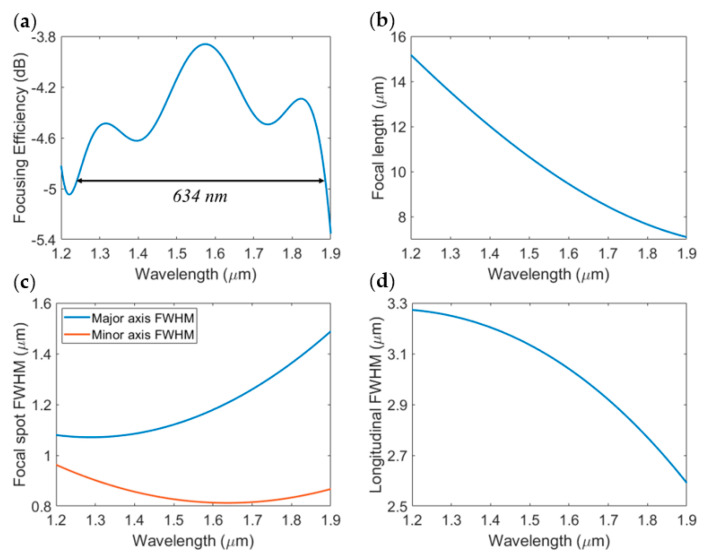
Wavelength dependence of (**a**) focal length, (**b**) focal spot transversal FWHM on major (blue) and minor (orange) axes, (**c**) focal spot longitudinal FWHM, and (**d**) the focusing efficiency of our proposed BPFZP with varying SWG duty cycle.

Once again, the focusing properties of the lens can be tuned by wavelength scanning. The spectral response of the focusing efficiency is plotted in Figure 12a. Based on our simulations, the 1 dB bandwidth was predicted to be 634 nm centered at 1.56 µm for the linear polarization, a 62% increase in bandwidth compared to the BPFZP design with a constant duty cycle. This translates to a focal length ranging from 7.1 µm to 15.2 µm, as shown in Figure 12b. The focal point maintains a subwavelength transverse FWHM on both the major and minor axes and has less than 700 nm variation in the longitudinal FWHM across the entire bandwidth, as plotted in Figure 12c,d, respectively.

### 3.3. Fabrication Tolerance

The effect of fabrication variation on the focusing performances of both designs was investigated. A possible fabrication error of +/− 5% in the SWG segment dimensions was considered. In both cases, the focal length and focal spot FWHMs remained constant relative to the nominal results. Our models predicted that the focusing efficiency of the polarization-insensitive and polarization-specific designs could be reduced by up to 5–7% compared to the maximum efficiency due to fabrication variations. However, this represents the extreme case where all dimensions in the lens have a +/−5% error. In practice, we expect the focusing efficiency to be closer to the nominal results with reasonable fabrication variations.

## 4. Conclusions

The design and simulation of both polarization-insensitive and polarization-specific 500 nm SOI-compatible binary phase Fresnel zone plate lenses with one single etch fabrication were presented. Subwavelength focusing capabilities with a wavelength-tunable focal distance and wide bandwidths of 389 nm and 634 nm were presented for the polarization-insensitive and linear polarization designs, respectively. A near-diffraction-limited circular focal spot with a FWHM of 0.64 λ and focusing efficiency of 39.2% were achieved with the circular polarization. Under the influence of both linear polarizations, an elliptical focal point with FWHMs of 0.84 λ and 0.53 λ along the major and minor axes, respectively, and focusing efficiency of 35.9% were demonstrated. The focusing efficiency and focal spot parameters are comparable with, if not better than, those previously reported in the literature, with the key advantage being the ease of fabrication due to the SOI compatibility. By optimizing the SWG duty cycle for a specific linear polarization, the focusing efficiency could be increased to 45.2% while reducing the major axis FWHM to 0.75 λ and increasing the 1 dB bandwidth by 62%. The wavelength-tunable focal distance of the lenses presented in this work is of particular interest for three-dimensional imaging. The size of the Fresnel zones can easily be modified to change the focal length and center wavelength for imaging and sensing applications with larger geometries. This is the first demonstration of a metalens fabricated on a standard SOI platform capable of producing a subwavelength focal spot size over a wide bandwidth. We believe that this work has applications in microscopy, medical imaging, and spatial multiplexing in free-space optics.

## Figures and Tables

**Table 1:** Comparison of results with previously reported metalenses.

Reference	Numerical Aperture	Focusing Efficiency	Bandwidth
This work	0.83	39.2%	1486–1874 nm
[9]	0.05	9.8%, 10.3%, 12.6%	1300 nm, 1550 nm, 1800 nm
[12]	0.1–0.24	77.1–88.5%	650–1000 nm
[15]	0.28	-	1250–1370 nm
[26]	0.2	26%	532 nm
[26]	0.97	13%	532 nm
[26]	0.97	4%	473 nm, 532 nm, 633 nm
[44]	0.59	4.1%	633 nm
[45]	0.24	≤60%	1300–1650 nm
[45]	0.24	≤50%	1200–1650 nm
[45]	0.13	≤55%	1200–1650 nm
[45]	0.88	-	1200–1400 nm

## Data Availability

The datasets generated and/or analyzed during the current study are available from the corresponding author on reasonable request.
